# Astrocyte activation in the anterior cingulate cortex and altered glutamatergic gene expression during paclitaxel-induced neuropathic pain in mice

**DOI:** 10.7717/peerj.1350

**Published:** 2015-10-22

**Authors:** Willias Masocha

**Affiliations:** Department Pharmacology and Therapeutics, Faculty of Pharmacy, Kuwait University, Safat, Kuwait

**Keywords:** Neuropathic pain, Anterior cingulate cortex, Astrocyte, Paclitaxel, Glutamate, Glutamate receptors, Glutamate transporters

## Abstract

Spinal astrocyte activation contributes to the pathogenesis of paclitaxel-induced neuropathic pain (PINP) in animal models. We examined glial fibrillary acidic protein (GFAP; an astrocyte marker) immunoreactivity and gene expression of GFAP, glutamate transporters and receptor subunits by real time PCR in the anterior cingulate cortex (ACC) at 7 days post first administration of paclitaxel, a time point when mice had developed thermal hyperalgesia. The ACC, an area in the brain involved in pain perception and modulation, was chosen because changes in this area might contribute to the pathophysiology of PINP. GFAP transcripts levels were elevated by more than fivefold and GFAP immunoreactivity increased in the ACC of paclitaxel-treated mice. The 6 glutamate transporters (GLAST, GLT-1 EAAC1, EAAT4, VGLUT-1 and VGLUT-2) quantified were not significantly altered by paclitaxel treatment. Of the 12 ionotropic glutamate receptor subunits transcripts analysed 6 (GLuA1, GLuA3, GLuK2, GLuK3, GLuK5 and GLuN1) were significantly up-regulated, whereas GLuA2, GLuK1, GLuK4, GLuN2A and GLuN2B were not significantly altered and GLuA4 was lowly expressed. Amongst the 8 metabotropic receptor subunits analysed only mGLuR8 was significantly elevated. In conclusion, during PINP there is astrocyte activation, with no change in glutamate transporter expression and differential up-regulation of glutamate receptor subunits in the ACC. Thus, targeting astrocyte activation and the glutamatergic system might be another therapeutic avenue for management of PINP.

## Introduction

The anterior cingulate cortex (ACC) is a cortical area in the brain that has been described to be involved with pain, possibly including both perception and modulation (Vogt, 2005; Xie, Huo & Tang, 2009; Zhuo, 2008). It is a component of the medial pain pathway. The afferent inputs to the ACC are from midline and intralaminar thalamic nuclei, whilst the ACC sends projections into various areas including the intralaminar thalamic nuclei and periaqueductal grey (PAG, which is involved in control of descending pain) (Senapati et al., 2005; Sewards & Sewards, 2002; Vogt, 2005). Neuroimaging studies have shown increased activity in the ACC during chronic pain, including neuropathic pain (Hsieh et al., 1995; Peyron, Laurent & Garcia-Larrea, 2000; Tseng et al., 2013). Neurophysiological and molecular changes have also been observed in the ACC during chronic or neuropathic (Wrigley et al., 2009; Xu et al., 2008; Yamashita et al., 2014).

One of the changes that has been observed in the ACC during chronic or neuropathic pain is the activation of astrocytes or astrogliosis (Chen et al., 2012; Kuzumaki et al., 2007; Lu, Zhu & Gao, 2011; Narita et al., 2006; Yamashita et al., 2014). Astrocytes are the most numerous non-neuronal cells in the brain involved in modulation of neuronal activities e.g., extracellular and synaptic cleft neurotransmitter level regulation, release of neuroactive molecules amongst other activities (Maragakis & Rothstein, 2006; Seifert, Schilling & Steinhauser, 2006). Astrocytes express transporters which remove neurotransmitters such as *γ*-aminobutyric acid (GABA) and glutamate from the extracellular space or synaptic cleft (Conti et al., 1998; Danbolt, 2001; Gosselin, Bebber & Decosterd, 2010; Minelli et al., 1995; Wang & Bordey, 2008). Astrocyte activation has been linked with increase in transporters for GABA and a decrease in transporters for glutamate resulting in a more excitatory state in the brain (Gosselin, Bebber & Decosterd, 2010; Maragakis & Rothstein, 2006). Recently, we observed an increase in the transcripts of GABA transporter 1 (GAT-1) in a rodent model of paclitaxel-induced neuropathic pain (PINP) (Masocha, 2015). However, it is not known whether paclitaxel induces astrocyte activation in the ACC although it has been shown to induce astrocyte activation in the spinal cord (Peters et al., 2007; Zhang, Yoon & Dougherty, 2012). Paclitaxel is a chemotherapeutic agent that causes dose-dependent neuropathic pain in some patients (Scripture, Figg & Sparreboom, 2006; Wolf et al., 2008). In the rodent models, we observed that the PINP is linked with disturbances in the GABAergic system (Masocha, 2015) resulting in increased excitability of the ACC to electrophysiological stimulation (H Nashawi, IO Edafiogho, SB Kombian, W Masocha, 2015, unpublished data). GABA is the major inhibitory neurotransmitter while glutamate is the major stimulatory neurotransmitter in the brain (Meldrum, 2000; Petroff, 2002). It is not known whether paclitaxel causes any changes in the glutamatergic system in the ACC, although it has been shown to decrease the expression of glutamate transporters such as GLAST and GLT-1 in the spinal cord (Weng et al., 2005; Zhang, Yoon & Dougherty, 2012). There are 8 known glutamate transporters, which are excitatory amino acid transporter 1 (EAAT1; referred to as GLAST in rodents), EAAT2 (GLT-1), EAAT3 (EAAC1), EAAT4, EAAT5, vesicular glutamate transporter 1 (VGLUT1), VGLUT2, and VGLUT3 (Danbolt, 2001; Shigeri, Seal & Shimamoto, 2004). Of the transporters, GLAST and GLT-1 are expressed on astrocytes (Danbolt, 2001) and play an important role in removal of glutamate from the synaptic cleft and extracellular space (Danbolt, 2001; Shigeri, Seal & Shimamoto, 2004); if their expression is down-regulated, this results in increased levels of glutamate and excitotoxicity (Danbolt, 2001; Rothstein et al., 1996; Shigeri, Seal & Shimamoto, 2004; Yi, Pow & Hazell, 2005). Glutamate acts on ionotropic and metabotropic receptors. The ionotropic receptors are divided into alpha-amino-3-hydroxy-5-methyl-4-isoxazolpropionate (AMPA), kainate and N-methyl-D-aspartate (NMDA) receptors which have 18 subunits GLuA1 to 4, GLuK1-5 and GLuN1, GLuN2A to D, GLuN3A and B, and GLuD1 and 2 (Collingridge et al., 2009). There are 8 subunits of the metabotropic receptors mGLUR_1_ to _8_ (Conn & Pin, 1997; Niswender & Conn, 2010).

Astrocyte activation, which has been observed in the ACC in models of chronic and neuropathic pain (Chen et al., 2012; Kuzumaki et al., 2007; Lu, Zhu & Gao, 2011; Narita et al., 2006; Yamashita et al., 2014), might occur in the ACC during PINP together with molecular changes in the glutamatergic system contributing to the pathogenesis or maintenance of PINP. Thus, in this study, astrocyte activation and the gene expression of molecules of the astrocyte marker (glial fibrillary acidic protein (GFAP), glutamate transporters and receptors in the ACC were evaluated in mice at a time point when the mice had paclitaxel-induced thermal hyperalgesia (Nieto et al., 2008; Parvathy & Masocha, 2013).

## Materials and Methods

### Animals

Ninety eight female BALB/c mice (8–12 weeks old) supplied by the Animal Resources Centre (ARC) at the Health Sciences Center (HSC), Kuwait University were used. The animals were housed and handled in compliance with the Kuwait University, HSC, ARC guidelines and published ethical guidelines for research in experimental pain with conscious animals (Zimmermann, 1983). All animal experiments were approved by the Ethical Committee for the use of Laboratory Animals in Teaching and in Research, HSC, Kuwait University.

### Paclitaxel administration

Paclitaxel (Cat. No. 1097; Tocris, Bristol, UK) was dissolved in a solution made up of 50% Cremophor EL and 50% absolute ethanol to a concentration of 6 mg/ml and then diluted in normal saline (NaCl 0.9%), to a final concentration of 0.2 mg/ml just before administration. The vehicle for paclitaxel, thus, constituted of about 1.7% Cremophor EL and 1.7% ethanol in normal saline. Paclitaxel 2 mg/kg or its vehicle were administered to mice intraperitoneally (i.p.), daily for 5 consecutive days. This treatment regimen has been reported to produce painful neuropathy and thermal hyperalgesia in mice (Nieto et al., 2008; Parvathy & Masocha, 2013).

### Hot plate test

Reaction latencies to hot plate test were measured before (baseline latency) and on day 7 after first administration of paclitaxel. Briefly, mice were placed on a hot plate (Panlab SL, Barcelona, Spain) with the temperature adjusted to 55 ± 1 °C. The time to the first sign of nociception, paw licking or flinching, was recorded and the animal immediately removed from the hot plate. A cut-off period of 20 s was maintained to avoid damage to the paws.

### ACC tissue preparation

The mice were anesthetized with isoflurane and sacrificed by decapitation. ACC was dissected and prepared for RNA extraction on day 7 post-first administration of paclitaxel—a time point when mice had developed thermal hyperalgesia (Parvathy & Masocha, 2013)—as previously described (Masocha, 2015).

### Real time RT-PCR

Gene transcripts of the astrocyte marker GFAP, 6 glutamate transporters (GLAST, GLT-1, EAAC1, EAAT4, VGLUT1, VGLUT2), 12 ionotropic glutamate receptor subunits (GLuA1 to 4, GLuK1 to 5, GLuN1, GLuN2A and GLuN2B) and 8 metabotropic glutamate subunits (mGluR_1_ to _8_) were quantified in the ACC of vehicle-treated or paclitaxel-treated by real time PCR. Total RNA was extracted from the fresh frozen ACC using the RNeasy Kit (Qiagen GmbH, Venlo, Netherlands), reverse-transcribed, and the mRNA levels were quantified on an ABI Prism^®^ 7500 sequence detection system (Applied Biosystems, Carlsbad, California, USA) as previously described (Masocha, 2009). The primer sequences which were used, listed in Table 1, were ordered from Invitrogen (Life Technologies, Carlsbad, California, USA) and/or synthesized at the Research Core Facility (RCF), HSC, Kuwait University. The amplification and detection were performed as follows: a first hold at 50 °C for 2 min, a second hold at 95 °C for 2 min followed by 40 cycles at 95 °C for 15 s and 63 °C for 1 min. Threshold cycle (Ct) values for all cDNA samples were obtained and the amount of mRNA of individual animal sample (*n* = 6–24 per group) was normalized to cyclophilin (housekeeping gene) (ΔCt). The relative amount of target gene transcripts was calculated using the 2^−ΔΔCt^ method as described previously (Livak & Schmittgen, 2001).

**Table 1:** PCR primer sequences of cyclophilin, GFAP and glutamatergic system molecules.

Gene	Polarity	
	Sense Sequence 5′–3′	Anti-sense Sequence 5′–3′
Cyclophilin	GCTTTTCGCCGCTTGCT	CTCGTCATCGGCCGTGAT
GFAP	ACAGCGGCCCTGAGAGAGAT	CTCCTCTGTCTCTTGCATGTTACTG
GLAST	ACCAAAAGCAACGGAGAAGAG	GGCATTCCGAAACAGGTAACTC
GLT-1	ACAATATGCCCAAGCAGGTAGA	CTTTGGCTCATCGGAGCTGA
EAAC1	CTTCCTACGGAATCACTGGCT	CGATCAGCGGCAAAATGACC
EAAT4	AGCAGCCACGGCAATAGTC	ATGCCAAGCTGACACCAATGA
VGLUT-1	GGTGGAGGGGGTCACATAC	AGATCCCGAAGCTGCCATAGA
VGLUT-2	CCCTGGAGGTGCCTGAGAA	GCGGTGGATAGTGCTGTTGTT
GLuA1	CCGTTGACACATCCAATCAGTTT	GTCGATAATGCTAATGAGAGCTTCCT
GLuA2	AAATTGCCAAACATTGTGG	ATGGAGCCATGGCAATATCA
GLuA3	ACACCATCAGCATAGGTGGA	TCAGTGGTGTTCTGGTTGGT
GLuA4	TTGGAATGGGATGGTAGGAG	TAGGAACAAGACCACGCTGA
GLuK1	TCACACCCTACGAGTGGTATAAC	AGCTCCAACGCCAAACCAG
GLuK2	ATCGGATATTCGCAAGGAACC	CCATAGGGCCAGATTCCACA
GLuK3	AGGTCCTAATGTCACTGACTCTC	GCCATAAAGGGTCCTATCAGAC
GLuK4	CCAAGGTCGAAGTGGACATCT	CTGGGGTGAAGGTTCAGGG
GLuK5	ATAGTCGCCTTCGCCAATCC	GTGTCCGTGGTCTCGTACTG
GLuN1	GGCATCGTAGCTGGGATCTTC	TCCTACGGGCATCCTTGTG
GLuN2A	GTTTGTTGGTGACGGTGAGA	AAGAGGTGCTCCCAGATGAA
GLuN2B	ATGTGGATTGGGAGGATAGG	TCGGGCTTTGAGGATACTTG
mGluR1	TGTCATCAACGCCATCTATGC	CCCACGTAGCCAGGACATAGAG
mGluR2	CGCTCTCTGCACGCTCTATG	GATGAACTTGGCCTCGTTGAA
mGluR3	AAGCCATCGCCTGTCATCTG	GGAGGTCCCAAGCCCAAGT
mGluR4	GATGCTCTACATGCCCAAAGTCTAC	CGGTGACAACGGCTTTGAG
mGluR5	TGACCCTGAGCCCATTGC	AACGAAGAGGGTGGCTAGCA
mGluR6	TCATGGCCACCACAACTATCA	CAGAGGCGCGGACTATGG
mGluR7	AAGCCTGGGCAGAGGAAGA	TCCATCACAGGGCTCACAAG
mGluR8	CAGCATCTGTCTGCAGCCTG	CGGTTTTCTTCCTCTCCCCA

### Immunohistochemistry

Fresh-frozen brains were cut on a cryostat into 25 µm thick sections and thaw-mounted on chrome-alum gelatin–coated slides. The sections at a level of the lateral ventricles and the ACC were fixed in 4% formalin and 14% picric acid in PBS for 30 s at 4 °C, rinsed in PBS, fixed in acetone for 30 s at −20 °C, and then rinsed in PBS. All sections were preincubated with 1% bovine serum albumin and 0.3% Triton X-100 in PBS (solution used as diluent for primary and secondary antibodies) for 30 min at room temperature. Sections were incubated with rabbit anti- GFAP (1:100; DAKO, Glostrup, Denmark) for 2 h at room temperature to immunostain astrocytes. Sections were then rinsed in PBS and incubated with DyLight 594-conjugated Affinipure donkey Anti-rabbit IgG (H + L) (1:100, Jackson ImmunoResearch Laboratories, West Grove, Pennsylvania, USA) for 1 h. The sections were rinsed in PBS and mounted in ProLong^®^ Gold antifade reagent (Invitrogen, USA). Sections were examined and analysed using a LSM 700 laser scanning confocal microscope. Images were taken from the ACC using an Axio imager (Carl Zeiss MicroImaging GmbH, Oberkochen, Germany).

### Statistical analyses

Statistical analyses were performed using unpaired two-tailed Student’s *t*-test using Graph Pad Prism software (version 5.0). The differences were considered significant at *p* < 0.05. The results in the text and figures are expressed as the means ± S.E.M.

## Results

### Paclitaxel-induced thermal hyperalgesia

Mice developed thermal hyperalgesia on day 7 after first administration of paclitaxel as we previously described (Masocha, 2014; Parvathy & Masocha, 2013) i.e., paclitaxel-treated mice had significant reduction in response latency time in the hot plate test on day 7 compared to the baseline latency and vehicle-treated animals (6.23 ± 0.28 s compared to 9.66 ± 0.16 s and 9.00 ± 0.38 s, respectively; *n* = 10 vehicle-treated mice and 16 paclitaxel treated-mice; *p* < 0.05 for both comparisons).

### Astrocyte activation in the ACC at 7 days after paclitaxel administration

**Figure 1:**
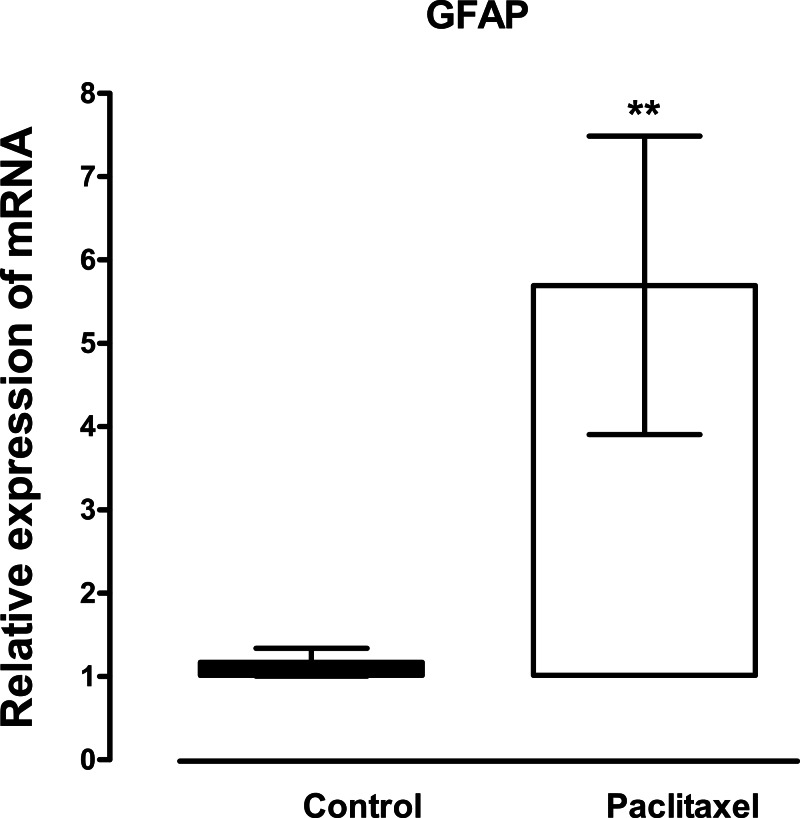
Effects of paclitaxel on glial fibrillary acidic protein (GFAP) transcript levels in the anterior cingulate cortex (ACC). Relative GFAP mRNA expression in the ACC of BALB/c mice on day 7 after first administration of the drug or its vehicle. Each point represents the mean ± S.E.M of the values obtained from 21 vehicle-treated control mice and 24 paclitaxel-treated mice. ^∗∗^ *p* < 0.01 compared to vehicle-treated control mice.

**Figure 2:**
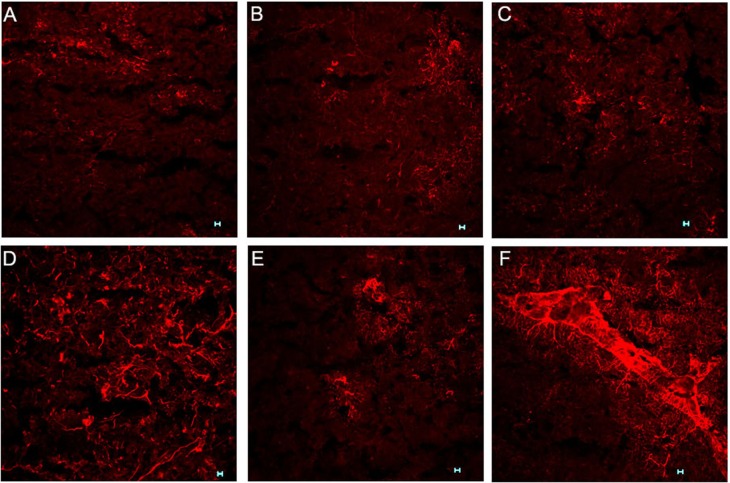
Effects of paclitaxel on glial fibrillary acidic protein (GFAP) immunoreactivity in the anterior cingulate cortex (ACC). GFAP immunoreactivity in the ACC of BALB/c mice on day 7 after first administration of the drug or its vehicle. GFAP immunoreactivity in astrocytes is increased in 3 paclitaxel-treated mice (D–F) compared to 3 vehicle-treated control mice (A–C) in the ACC. Note that in a paclitaxel-treated mouse (D) increased immunoreactivity of GFAP appears to be along a blood vessel: Scale bar: 50 µm.

The mRNA expression and immunoreactivity of the astrocyte marker, GFAP, were analysed in the ACC at day 7, a time when the mice had developed thermal hyperalgesia. Treatment with paclitaxel significantly increased the expression of GFAP transcripts (*p* = 0.02) by more than fivefold compared to vehicle-treated controls (Fig. 1). Confocal microscopy images showed that in paclitaxel-treated mice there was increased GFAP immunoreactivity in the ACC compared to vehicle-treated controls (Fig. 2). However, the change in GFAP immunoreactivity in paclitaxel-treated animals varied across the ACC and animals i.e., it was not robust in all animals and did not cover most of the ACC.

### Expression of transcripts of glutamate transporters in the ACC at 7 days after paclitaxel administration

**Figure 3:**
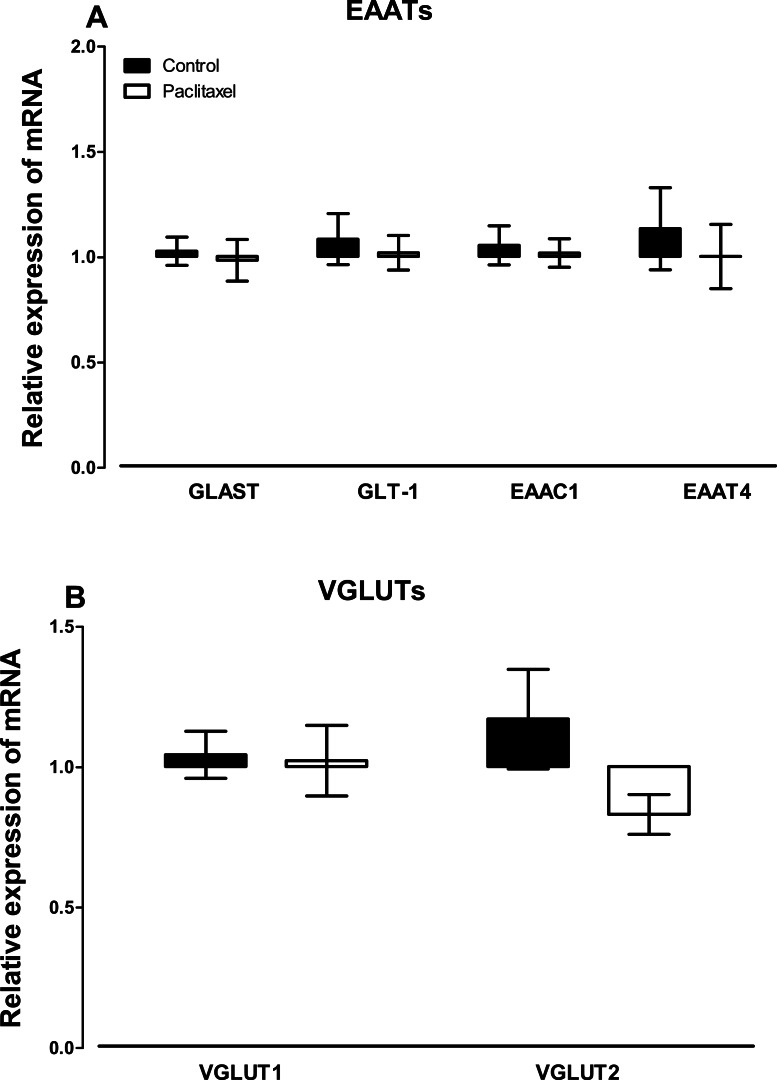
Effects of paclitaxel on glutamate transporters transcript levels in the anterior cingulate cortex (ACC). Relative mRNA expression of (A) excitatory amino acid transporters GLAST, GLT-1, EAAC1, EAAT4, and (B) vesicular glutamate transporters VGLUT1 and VGLUT2 in the ACC of BALB/c mice on day 7 after first administration of the drug or its vehicle. Each point represents the mean ± S.E.M of the values obtained from 11 to 15 vehicle-treated control mice and 13–15 paclitaxel-treated mice.

There were no differences observed in the transcript levels of all the six glutamate transporters analysed (Fig. 3) in the ACC of paclitaxel-treated mice compared to vehicle-treated mice. Using the unpaired two-tailed Student’s *t*-test the *p* values obtained are: 0.7243 for GLAST, 0.6608 for GLT-1, 0.7575 for EAAC1, 0.5925 for EAAT4, 0.8885 for VGLUT-1 and 0.0858 for VGLUT-2.

### Expression of transcripts of glutamate receptors in the ACC at 7 days after paclitaxel administration

**Figure 4:**
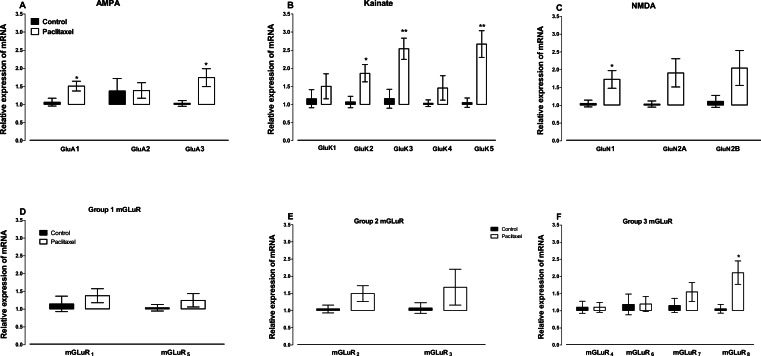
Effects of paclitaxel on glutamate receptors transcript levels in the anterior cingulate cortex (ACC). Relative mRNA expression of (A) AMPA receptor subunits GLuA1 to 3, (B) kainate receptor subunits GLuK1 to 5, (C) NMDA receptor subunits GLuN1, GLuN2A and GLuN2B, and (D-F) metabotropic glutamate receptors mGLuR_1_ to _8_ in the ACC of BALB/c mice on day 7 after first administration of the drug or its vehicle. Each point represents the mean ± S.E.M of the values obtained from 6 to 15 vehicle-treated control mice and 8–16 paclitaxel-treated mice. ^∗^*p* < 0.05, ^∗∗^*p* < 0.01 compared to vehicle-treated control mice.

Amongst the AMPA receptor subunits GLuA4 was lowly expressed in the ACC and mRNA expression was not detected after 40 cycles in the real time RT-PCR in 12 out of 16 vehicle- and paclitaxel-treated animals analysed. Treatment with paclitaxel did not significantly alter the mRNA expression of the AMPA receptor subunit GLuA2 (*p* = 0.9720), but significantly increased the expression of GLuA1 (*p* = 0.0166) and GLuA3 (*p* = 0.0243) subunits compared to vehicle-treated controls (Fig. 4A).

Amongst the 5 kainate receptor subunits analysed, treatment with paclitaxel significantly increased the expression of the 3 subunits GluK2 (*p* = 0.0136), GluK3 (*p* = 0.0026) and GluK5 (*p* = 0.0011), but not 2 subunits GluK1 (*p* = 0.4367) and GluK4 (*p* = 0.2785), compared to vehicle-treated controls (Fig. 4B).

Amongst the 3 NMDA receptor subunits analysed, treatment with paclitaxel significantly increased the expression of GluN1 (*p* = 0.0209) only, but not 2 subunits GluN2A (*p* = 0.0612) and GluN2B (*p* = 0.1105), compared to vehicle-treated controls (Fig. 4C).

Of all the eight metabotropic glutamate receptors subunits quantified, only mGLuR_8_ was significantly altered (*p* = 0.0144) in the ACC by treatment with paclitaxel compared to treatment with vehicle (Figs. 4E and 4F). Using the unpaired two-tailed Student’s *t*-test, the *p* values obtained are: 0.4439 for mGLuR_1_, 0.1340 for mGLuR_2_, 0.3201 for mGLuR_3_, 0.9971 for mGLuR_4_, 0.3375 for mGLuR_5_, 0.9693 for mGLuR_6_ and 0.2780 for mGLuR_7_.

## Discussion

This is the first study to report on the quantification and/or changes in the transcript levels and immunoreactivity of the astrocyte marker GFAP, transcript levels of glutamate transporters and receptors in the ACC, an area associated with pain perception and modulation (Vogt, 2005; Xie, Huo & Tang, 2009; Zhuo, 2008), during paclitaxel-induced neuropathic pain (PINP).

Increased expression of GFAP in the brain is a marker of astrocyte activation (Aldskogius & Kozlova, 1998). Various studies have reported increased expression of GFAP mRNA and protein in the ACC during pain (Chen et al., 2012; Kuzumaki et al., 2007; Lu, Zhu & Gao, 2011). Astrocyte activation has also been observed in the ACC in other models of neuropathic pain (Xu et al., 2008; Yamashita et al., 2014) but had not been reported in PINP. However, astrocyte activation in the spinal cord has been reported to contribute to PINP in rodents (Ruiz-Medina et al., 2013; Zhang, Yoon & Dougherty, 2012). In the current study, the expression of GFAP transcripts and immunoreactivity in the ACC was increased in mice treated with PINP. During peripheral nerve injury neurons have been reported to release neurotransmitters such substance *P* and glutamate and neuronal chemokines that cause astrocyte activation in the CNS (Milligan & Watkins, 2009; Wang et al., 2009; Watkins et al., 2007). Activated astrocytes in turn release molecules that contribute to the pathophysiology of pain through modulation of neuronal functioning (Milligan & Watkins, 2009; Wang et al., 2009; Watkins et al., 2007). Thus, the current results suggest that astrocyte activation in the ACC might also contribute to the pathophysiology of PINP.

The activation of astrocytes in the spinal cord induced by paclitaxel has been reported to be accompanied with a decrease in the expression of the glial glutamate transporters GLAST and GLT-1 (Zhang, Yoon & Dougherty, 2012) as well as an increase in the GABA transporter GAT-1 (Yadav et al., 2015). In the current study, there were no changes in the transcript levels of glutamate transporters in the ACC of paclitaxel-treated mice. However, in a recent study, we observed elevated transcripts of GAT-1 in the ACC of mice with PINP (Masocha, 2015). This suggests that astrocyte activation and increased expression of GAT-1, but not glutamate transporters, in the ACC play a role in the pathogenesis in PINP. This would result in an imbalance in the inhibitory (GABA) and excitatory (glutamate) neurotransmitters, which might result in increased excitability of the ACC. Increased neuronal excitability in the ACC might contribute to the increased activity observed in the ACC during neuropathic pain in both humans and animal models (Hsieh et al., 1995; Peyron, Laurent & Garcia-Larrea, 2000; Tseng et al., 2013; Wrigley et al., 2009; Xu et al., 2008; Yamashita et al., 2014).

Although we did not observe any changes in the glutamate transporters in the ACC, we observed that transcripts of various glutamate receptors and receptor subunits were elevated in the ACC of mice with PINP. The increased expression of some of the glutamate receptors and receptor subunits could have been linked to astrocyte activation since all of the up-regulated receptors are expressed on astrocytes (Geurts et al., 2005; Martínez-Lozada & Ortega, 2015). Several receptors have been reported to be differentially expressed in the ACC in rodent models of PINP. We observed an increase in the expression of various GABA receptors in the ACC during PINP (Masocha, 2015). Ortega-Legaspi et al. (2011) and Ortega-Legaspi et al. (2010). reported a differential expression of muscarinic-1 and −2 receptors and dopamine D1 and D2 receptors in the ACC of rodents with PINP. The increased expression glutamate receptors in the ACC also suggest a role of the glutamatergic system in the pathogenesis of PINP.

## Conclusions

In conclusion, the results of this study show that animals with paclitaxel-induced neuropathic pain (PINP) have increased transcripts and immunoreactivity of the astrocyte marker GFAP and transcripts of some glutamate receptors and receptor subunits, but not glutamate transporters, in the ACC. In a previous study, transcripts of a GABA transporter GAT-1, whose increase has been associated with astrocyte activation in the spinal cord of rodents with PINP (Yadav et al., 2015), was found increased in the ACC of mice with PINP (Masocha, 2015). Thus, inhibition of astrocyte activation and GAT-1 activity and/or antagonism of specific glutamate receptors could be therapeutic modalities of managing PINP and possibly other types on chemotherapy-induced neuropathic pain.

## Supplemental Information

10.7717/peerj.1350/supp-1 Supplemental Information 1 Raw data—Relative expression of mRNA for GFAP

10.7717/peerj.1350/supp-2 Supplemental Information 2 Raw data—Relative expression of mRNA for glutamate transporters

10.7717/peerj.1350/supp-3 Supplemental Information 3 Raw data—Relative expression of mRNA for kainate glutamate receptor subunits

10.7717/peerj.1350/supp-4 Supplemental Information 4 Raw data—Relative expression of mRNA for kainate glutamate receptor subunits

10.7717/peerj.1350/supp-5 Supplemental Information 5 Raw data—Relative expression of mRNA for NMDA glutamate receptor subunits

10.7717/peerj.1350/supp-6 Supplemental Information 6 Raw data—Relative expression of mRNA for metabotropic glutamate receptor

10.7717/peerj.1350/supp-7 Supplemental Information 7 Raw data—Effects of paclitaxel on glial fibrillaryacidic protein (GFAP) immunoreactivity in the anterior cingulate cortex (ACC) on vehicle-treated mouse number 1

10.7717/peerj.1350/supp-8 Supplemental Information 8 Raw data—Effects of paclitaxel on glial fibrillary acidic protein (GFAP) immunoreactivity in the anterior cingulate cortex (ACC) on vehicle-treated mouse number 2

10.7717/peerj.1350/supp-9 Supplemental Information 9 Raw data—Effects of paclitaxel on glial fibrillary acidic protein (GFAP) immunoreactivity in the anterior cingulate cortex (ACC) on vehicle-treated mouse number 3

10.7717/peerj.1350/supp-10 Supplemental Information 10 Raw data—Effects of paclitaxel on glial fibrillary acidic protein (GFAP) immunoreactivity in the anterior cingulate cortex (ACC) on paclitaxel-treated mouse number 1

10.7717/peerj.1350/supp-11 Supplemental Information 11 Raw data—Effects of paclitaxel on glial fibrillary acidic protein (GFAP) immunoreactivity in the anterior cingulate cortex (ACC) on paclitaxel-treated mouse number 2

10.7717/peerj.1350/supp-12 Supplemental Information 12 Raw data—Effects of paclitaxel on glial fibrillary acidic protein (GFAP) immunoreactivity in the anterior cingulate cortex (ACC) on paclitaxel-treated mouse number 3

## References

[ref-1] Aldskogius H, Kozlova EN (1998). Central neuron-glial and glial–glial interactions following axon injury. Progress in Neurobiology.

[ref-2] Chen FL, Dong YL, Zhang ZJ, Cao DL, Xu J, Hui J, Zhu L, Gao YJ (2012). Activation of astrocytes in the anterior cingulate cortex contributes to the affective component of pain in an inflammatory pain model. Brain Research Bulletin.

[ref-3] Collingridge GL, Olsen RW, Peters J, Spedding M (2009). A nomenclature for ligand-gated ion channels. Neuropharmacology.

[ref-4] Conn PJ, Pin JP (1997). Pharmacology and functions of metabotropic glutamate receptors. Annual Review of Pharmacology and Toxicology.

[ref-5] Conti F, Melone M, De Biasi S, Minelli A, Brecha NC, Ducati A (1998). Neuronal and glial localization of GAT-1, a high-affinity gamma-aminobutyric acid plasma membrane transporter, in human cerebral cortex: with a note on its distribution in monkey cortex. Journal of Comparative Neurology.

[ref-6] Danbolt NC (2001). Glutamate uptake. Progress in Neurobiology.

[ref-7] Geurts JJ, Wolswijk G, Bo L, Redeker S, Ramkema M, Troost D, Aronica E (2005). Expression patterns of Group III metabotropic glutamate receptors mGluR4 and mGluR8 in multiple sclerosis lesions. Journal of Neuroimmunology.

[ref-8] Gosselin RD, Bebber D, Decosterd I (2010). Upregulation of the GABA transporter GAT-1 in the gracile nucleus in the spared nerve injury model of neuropathic pain. Neuroscience Letters.

[ref-9] Hsieh JC, Belfrage M, Stone-Elander S, Hansson P, Ingvar M (1995). Central representation of chronic ongoing neuropathic pain studied by positron emission tomography. Pain.

[ref-10] Kuzumaki N, Narita M, Hareyama N, Niikura K, Nagumo Y, Nozaki H, Amano T, Suzuki T (2007). Chronic pain-induced astrocyte activation in the cingulate cortex with no change in neural or glial differentiation from neural stem cells in mice. Neuroscience Letters.

[ref-11] Livak KJ, Schmittgen TD (2001). Analysis of relative gene expression data using real-time quantitative PCR and the 2(-Delta Delta C(T)) Method. Methods.

[ref-12] Lu Y, Zhu L, Gao YJ (2011). Pain-related aversion induces astrocytic reaction and proinflammatory cytokine expression in the anterior cingulate cortex in rats. Brain Research Bulletin.

[ref-13] Maragakis NJ, Rothstein JD (2006). Mechanisms of Disease: astrocytes in neurodegenerative disease. Nature Clinical Practice Neurology.

[ref-14] Martínez-Lozada Z, Ortega A (2015). Glutamatergic transmission: a matter of three. Neural Plasticity.

[ref-15] Masocha W (2009). Systemic lipopolysaccharide (LPS)-induced microglial activation results in different temporal reduction of CD200 and CD200 receptor gene expression in the brain. Journal of Neuroimmunology.

[ref-16] Masocha W (2014). Paclitaxel-induced hyposensitivity to nociceptive chemical stimulation in mice can be prevented by treatment with minocycline. Scientific Reports.

[ref-17] Masocha W (2015). Comprehensive analysis of the GABAergic system gene expression profile in the anterior cingulate cortex of mice with Paclitaxel-induced neuropathic pain. Gene Expression.

[ref-18] Meldrum BS (2000). Glutamate as a neurotransmitter in the brain: review of physiology and pathology. Journal of Nutrition.

[ref-19] Milligan ED, Watkins LR (2009). Pathological and protective roles of glia in chronic pain. Nature Reviews Neuroscience.

[ref-20] Minelli A, Brecha NC, Karschin C, DeBiasi S, Conti F (1995). GAT-1, a high-affinity GABA plasma membrane transporter, is localized to neurons and astroglia in the cerebral cortex. Journal of Neuroscience.

[ref-21] Narita M, Kuzumaki N, Narita M, Kaneko C, Hareyama N, Miyatake M, Shindo K, Miyoshi K, Nakajima M, Nagumo Y, Sato F, Wachi H, Seyama Y, Suzuki T (2006). Chronic pain-induced emotional dysfunction is associated with astrogliosis due to cortical delta-opioid receptor dysfunction. Journal of Neurochemistry.

[ref-22] Nieto FR, Entrena JM, Cendan CM, Pozo ED, Vela JM, Baeyens JM (2008). Tetrodotoxin inhibits the development and expression of neuropathic pain induced by paclitaxel in mice. Pain.

[ref-23] Niswender CM, Conn PJ (2010). Metabotropic glutamate receptors: physiology, pharmacology, and disease. Annual Review of Pharmacology and Toxicology.

[ref-24] Ortega-Legaspi JM, De Gortari P, Garduno-Gutierrez R, Amaya MI, Leon-Olea M, Coffeen U, Pellicer F (2011). Expression of the dopaminergic D1 and D2 receptors in the anterior cingulate cortex in a model of neuropathic pain. Molecular Pain.

[ref-25] Ortega-Legaspi JM, Leon-Olea M, De Gortari P, Amaya MI, Coffeen U, Simon-Arceo K, Pellicer F (2010). Expression of muscarinic M1 and M2 receptors in the anterior cingulate cortex associated with neuropathic pain. European Journal of Pain.

[ref-26] Parvathy SS, Masocha W (2013). Matrix metalloproteinase inhibitor COL-3 prevents the development of paclitaxel-induced hyperalgesia in mice. Medical Principles and Practice.

[ref-27] Peters CM, Jimenez-Andrade JM, Kuskowski MA, Ghilardi JR, Mantyh PW (2007). An evolving cellular pathology occurs in dorsal root ganglia, peripheral nerve and spinal cord following intravenous administration of paclitaxel in the rat. Brain Research.

[ref-28] Petroff OA (2002). GABA and glutamate in the human brain. Neuroscientist.

[ref-29] Peyron R, Laurent B, Garcia-Larrea L (2000). Functional imaging of brain responses to pain. A review and meta-analysis (2000). Neurophysiologie Clinique.

[ref-30] Rothstein JD, Dykes-Hoberg M, Pardo CA, Bristol LA, Jin L, Kuncl RW, Kanai Y, Hediger MA, Wang Y, Schielke JP, Welty DF (1996). Knockout of glutamate transporters reveals a major role for astroglial transport in excitotoxicity and clearance of glutamate. Neuron.

[ref-31] Ruiz-Medina J, Baulies A, Bura SA, Valverde O (2013). Paclitaxel-induced neuropathic pain is age dependent and devolves on glial response. European Journal of Pain.

[ref-32] Scripture CD, Figg WD, Sparreboom A (2006). Peripheral neuropathy induced by paclitaxel: recent insights and future perspectives. Current Neuropharmacology.

[ref-33] Seifert G, Schilling K, Steinhauser C (2006). Astrocyte dysfunction in neurological disorders: a molecular perspective. Nature Reviews Neuroscience.

[ref-34] Senapati AK, Lagraize SC, Huntington PJ, Wilson HD, Fuchs PN, Peng YB (2005). Electrical stimulation of the anterior cingulate cortex reduces responses of rat dorsal horn neurons to mechanical stimuli. Journal of Neurophysiology.

[ref-35] Sewards TV, Sewards MA (2002). The medial pain system: neural representations of the motivational aspect of pain. Brain Research Bulletin.

[ref-36] Shigeri Y, Seal RP, Shimamoto K (2004). Molecular pharmacology of glutamate transporters, EAATs and VGLUTs. Brain Research Reviews.

[ref-37] Tseng MT, Chiang MC, Chao CC, Tseng WY, Hsieh ST (2013). fMRI evidence of degeneration-induced neuropathic pain in diabetes: enhanced limbic and striatal activations. Human Brain Mapping.

[ref-38] Vogt BA (2005). Pain and emotion interactions in subregions of the cingulate gyrus. Nature Reviews Neuroscience.

[ref-39] Wang DD, Bordey A (2008). The astrocyte odyssey. Progress in Neurobiology.

[ref-40] Wang W, Wang W, Mei X, Huang J, Wei Y, Wang Y, Wu S, Li Y (2009). Crosstalk between spinal astrocytes and neurons in nerve injury-induced neuropathic pain. PLoS ONE.

[ref-41] Watkins LR, Hutchinson MR, Ledeboer A, Wieseler-Frank J, Milligan ED, Maier SF (2007). Norman Cousins Lecture. Glia as the “bad guys”: implications for improving clinical pain control and the clinical utility of opioids. Brain, Behavior, and Immunity.

[ref-42] Weng HR, Aravindan N, Cata JP, Chen JH, Shaw AD, Dougherty PM (2005). Spinal glial glutamate transporters downregulate in rats with taxol-induced hyperalgesia. Neuroscience Letters.

[ref-43] Wolf S, Barton D, Kottschade L, Grothey A, Loprinzi C (2008). Chemotherapy-induced peripheral neuropathy: prevention and treatment strategies. European Journal of Cancer.

[ref-44] Wrigley PJ, Press SR, Gustin SM, Macefield VG, Gandevia SC, Cousins MJ, Middleton JW, Henderson LA, Siddall PJ (2009). Neuropathic pain and primary somatosensory cortex reorganization following spinal cord injury. Pain.

[ref-45] Xie YF, Huo FQ, Tang JS (2009). Cerebral cortex modulation of pain. Acta Pharmacologica Sinica.

[ref-46] Xu H, Wu LJ, Wang H, Zhang X, Vadakkan KI, Kim SS, Steenland HW, Zhuo M (2008). Presynaptic and postsynaptic amplifications of neuropathic pain in the anterior cingulate cortex. Journal of Neuroscience.

[ref-47] Yadav R, Yan X, Maixner DW, Gao M, Weng HR (2015). Blocking the GABA transporter GAT-1 ameliorates spinal GABAergic disinhibition and neuropathic pain induced by paclitaxel. Journal of Neurochemistry.

[ref-48] Yamashita A, Hamada A, Suhara Y, Kawabe R, Yanase M, Kuzumaki N, Narita M, Matsui R, Okano H, Narita M (2014). Astrocytic activation in the anterior cingulate cortex is critical for sleep disorder under neuropathic pain. Synapse.

[ref-49] Yi JH, Pow DV, Hazell AS (2005). Early loss of the glutamate transporter splice-variant GLT-1v in rat cerebral cortex following lateral fluid-percussion injury. Glia.

[ref-50] Zhang H, Yoon SY, Dougherty PM (2012). Evidence that spinal astrocytes but not microglia contribute to the pathogenesis of Paclitaxel-induced painful neuropathy. The Journal of Pain.

[ref-51] Zhuo M (2008). Cortical excitation and chronic pain. Trends in Neurosciences.

[ref-52] Zimmermann M (1983). Ethical guidelines for investigations of experimental pain in conscious animals. Pain.

