# Supplementary material for: Astrocyte activation in the anterior cingulate cortex and altered glutamatergic gene expression during paclitaxel-induced neuropathic pain in mice
Source: PeerJ. 2015 Oct 22;3:e1350. doi: 10.7717/peerj.1350 (PMC4627912; doi:10.7717/peerj.1350)
Supplement: Supplemental Information 1 [file peerj-03-1350-s001.docx]

**Relative expression of mRNA for GFAP**

| Animal number | Control | Paclitaxel-treated |
| --- | --- | --- |
|  | 1.776809 | 0.6106096 |
|  | 0.5159091 | 0.8727794 |
|  | 0.4725773 | 0.7552828 |
|  | 1.109612 | 0.4840704 |
|  | 0.8187277 | 1.259620 |
|  | 2.540987 | 0.8241938 |
|  | 0.6228903 | 4.175165 |
|  | 0.3471064 | 23.792750 |
|  | 1.265888 | 26.184060 |
|  | 3.653679 | 29.515820 |
|  | 1.269510 | 12.135400 |
|  | 0.808010 | 9.083510 |
|  | 0.974870 | 11.704430 |
|  | 1.795808 | 0.9543564 |
|  | 0.9216132 | 2.561609 |
|  | 0.674006 | 0.4385662 |
|  | 0.8964531 | 2.125251 |
|  | 1.354561 | 0.6984885 |
|  | 1.161086 | 1.075588 |
|  | 0.8830855 | 0.7087874 |
|  | 0.7200031 | 0.6126245 |
|  |  | 0.5838173 |
|  |  | 0.5609804 |
|  |  | 4.948873 |
