# Supplementary material for: Astrocyte activation in the anterior cingulate cortex and altered glutamatergic gene expression during paclitaxel-induced neuropathic pain in mice
Source: PeerJ. 2015 Oct 22;3:e1350. doi: 10.7717/peerj.1350 (PMC4627912; doi:10.7717/peerj.1350)
Supplement: Supplemental Information 2 [file peerj-03-1350-s002.docx]

| **Transporter** | **Animal number** | **1** | **2** | **3** | **4** | **5** | **6** | **7** | **8** | **9** | **10** | **11** | **12** | **13** | **14** | **15** |
| --- | --- | --- | --- | --- | --- | --- | --- | --- | --- | --- | --- | --- | --- | --- | --- | --- |
| GLAST | Control (Vehicle-treated) | 1.510752 | 1.011830 | 0.7391471 | 0.8850515 | 0.7631539 | 1.251070 | 1.489501 | 0.7031783 | 1.285511 | 0.8626068 | 1.014762 | 0.8886836 | 1.202210 | 0.9423962 | 0.8826452 |
|  | Paclitaxel-treated | 0.3643929 | 0.9334427 | 1.132478 | 0.346901 | 0.5307101 | 1.066977 | 0.700349 | 0.9570337 | 0.8888068 | 1.195357 | 0.9285651 | 1.275361 | 1.439637 | 1.383516 | 1.649512 |
| GLT-1 | Control (Vehicle-treated) | 0.3823304 | 1.023439 | 2.249948 | 1.135865 | 0.7823101 | 0.9874383 | 1.798128 | 0.7199303 | 1.534485 | 0.9275546 | 0.714169 | 0.9837776 | 0.940587 | 1.282759 | 0.8288119 |
|  | Paclitaxel-treated | 0.9836327 | 1.176601 | 1.182542 | 0.7040562 | 0.9328471 | 1.603487 | 0.5323742 | 1.374339 | 1.036489 | 1.197150 | 1.046874 | 0.5282587 | 1.345414 | 0.6299241 | 1.043948 |
| \| EAAC1 \| \| --- \| \|  \| | Control (Vehicle-treated) | 0.5297821 | 1.154035 | 1.721267 | 0.9502446 | 0.6957062 | 1.362982 | 1.433293 | 0.7357818 | 1.542224 | 0.8449449 | 0.7449141 | 1.030191 | 1.422828 | 0.8165866 | 0.8606873 |
|  | Paclitaxel-treated | 1.043143 | 1.089277 | 0.9842778 | 0.4740354 | 0.9838771 | 1.131236 | 0.5023971 | 1.354408 | 1.012201 | 1.138759 | 1.402787 | 0.8067598 | 1.213727 | 1.143517 | 1.027433 |
| EAAT4 | Control (Vehicle-treated) | 1.000000 | 2.536392 | 0.9623128 | 0.4097013 | 2.195025 | 0.877206 | 0.5546838 | 0.9362966 | 1.178494 | 0.8598724 | 0.9868215 |  |  |  |  |
|  | Paclitaxel-treated | 1.822413 | 1.527229 | 0.2072932 | 0.9265203 | 0.2208662 | 0.8098093 | 0.8571211 | 1.229982 | 1.045798 | 0.4749764 | 1.739205 | 1.567604 | 0.6175072 |  |  |
| \| VGLUT1 \| \| --- \| | Control (Vehicle-treated) | 0.8885142 | 0.7646651 | 0.9254113 | 1.590485 | 0.8772212 | 1.024507 | 1.316337 | 0.8452964 | 1.012736 | 1.104499 | 0.5117786 | 1.746853 | 1.317051 | 0.8405435 | 0.9033108 |
|  | Paclitaxel-treated | 0.6172813 | 2.168103 | 1.308911 | 0.5412733 | 0.5804978 | 1.340864 | 0.5396375 | 1.859857 | 1.258910 | 0.8227779 | 0.6282127 | 0.9155397 | 0.838720 | 0.9601917 | 0.9675184 |
| \| VGLUT2 \| \| --- \| | Control (Vehicle-treated) | 0.2716586 | 2.669125 | 1.174028 | 1.174706 | 0.7384003 | 2.311119 | 1.126118 | 0.5203575 | 2.078901 | 0.7198263 | 0.6824464 | 0.9791968 | 1.405246 | 0.6827309 | 1.042313 |
|  | Paclitaxel-treated | 0.7190763 | 0.4364536 | 0.5570132 | 0.8605266 | 0.6273134 | 1.117642 | 0.8220654 | 0.6982848 | 0.3722441 | 1.300309 | 1.004066 | 0.7389194 | 1.112025 | 0.9740418 | 1.144610 |

|  |
| --- |
|  |
|  |

**Relative expression of mRNA for glutamate transporters**
