# Supplementary material for: Astrocyte activation in the anterior cingulate cortex and altered glutamatergic gene expression during paclitaxel-induced neuropathic pain in mice
Source: PeerJ. 2015 Oct 22;3:e1350. doi: 10.7717/peerj.1350 (PMC4627912; doi:10.7717/peerj.1350)
Supplement: Supplemental Information 3 [file peerj-03-1350-s003.docx]

| **Receptor** | **Animal number** | **1** | **2** | **3** | **4** | **5** | **6** | **7** | **8** | **9** | **10** | **11** | **12** | **13** | **14** | **15** | **16** |
| --- | --- | --- | --- | --- | --- | --- | --- | --- | --- | --- | --- | --- | --- | --- | --- | --- | --- |
| GluA1 | Control (Vehicle-treated) | 1.229144 | 0.7425041 | 0.9883685 | 0.795355 | 0.8026285 | 0.916149 | 2.041818 | 0.9283693 | 0.5567666 | 1.226356 | 1.464571 | 0.7281777 | 1.798045 | 1.017289 | 0.7507889 |  |
|  | Paclitaxel-treated | 1.291894 | 1.446154 | 1.199517 | 1.174750 | 1.092958 | 1.951956 | 0.9765647 | 1.561938 | 1.577377 | 1.379850 | 1.840964 | 1.727970 | 0.3755506 | 2.769315 | 1.714668 | 2.020267 |
| GluA2 | Control (Vehicle-treated) | 0.485321 | 1.312494 | 1.569906 | 0.567462 | 1.432593 | 2.011975 | 0.2717685 | 0.9889252 | 3.720810 |  |  |  |  |  |  |  |
|  | Paclitaxel-treated | 1.934679 | 1.508880 | 1.564908 | 1.439843 | 1.613652 | 1.810468 | 0.8833737 | 0.5853734 | 3.034061 | 1.331944 | 0.6963333 | 0.243760 |  |  |  |  |
| GluA3 | Control (Vehicle-treated) | 0.8190493 | 0.9641674 | 1.266303 | 0.6443483 | 1.342398 | 1.218210 | 0.9013823 | 0.9203011 | 1.205483 |  |  |  |  |  |  |  |
|  | Paclitaxel-treated | 2.966282 | 2.404503 | 2.560879 | 2.996106 | 0.6250005 | 1.025063 | 0.7912813 | 1.695150 | 1.354767 | 1.958765 | 1.705243 | 0.8466736 |  |  |  |  |

|  |
| --- |
|  |
|  |

**Relative expression of mRNA for kainate glutamate receptors subunits**
