# Supplementary material for: Astrocyte activation in the anterior cingulate cortex and altered glutamatergic gene expression during paclitaxel-induced neuropathic pain in mice
Source: PeerJ. 2015 Oct 22;3:e1350. doi: 10.7717/peerj.1350 (PMC4627912; doi:10.7717/peerj.1350)
Supplement: Supplemental Information 4 [file peerj-03-1350-s004.docx]

| **Receptor** | **Animal number** | **1** | **2** | **3** | **4** | **5** | **6** | **7** | **8** | **9** | **10** | **11** | **12** |
| --- | --- | --- | --- | --- | --- | --- | --- | --- | --- | --- | --- | --- | --- |
| GluK1 | Control (Vehicle-treated) | 1.498376 | 0.880320 | 0.758121 | 1.447041 | 0.8023511 | 0.8613007 | 0.6061233 | 3.215775 | 0.5153413 | 0.9955392 |  |  |
|  | Paclitaxel-treated | 0.8700076 | 1.317984 | 1.828423 | 0.7879631 | 1.154690 | 1.629699 | 0.957950 | 1.133360 | 1.364968 | 4.774832 | 0.6992919 |  |
| GluK2 | Control (Vehicle-treated) | 1.022720 | 1.008710 | 0.9693421 | 0.8769332 | 0.9945889 | 1.146542 | 0.6982828 | 2.432011 | 0.7479515 | 0.7872807 |  |  |
|  | Paclitaxel-treated | 0.7921494 | 1.915841 | 3.339760 | 1.624893 | 1.247701 | 2.710149 | 2.257572 | 2.243118 | 0.7649744 | 2.196211 | 1.422192 |  |
| GluK3 | Control (Vehicle-treated) | 0.8115146 | 1.019260 | 1.208979 | 0.8769332 | 0.9945889 | 1.146542 | 1.001593 | 3.427752 | 0.6803721 | 0.4281077 |  |  |
|  | Paclitaxel-treated | 2.750141 | 4.139593 | 3.915841 | 3.428369 | 1.247701 | 2.710149 | 2.257572 | 2.243118 | 1.771530 | 1.222976 | 2.250058 |  |
| GluK4 | Control (Vehicle-treated) | 0.993169 | 1.271952 | 0.7916005 | 1.324410 | 1.108839 | 0.6809402 | 1.413564 | 1.314111 | 0.6785314 | 0.793382 |  |  |
|  | Paclitaxel-treated | 2.000200 | 3.897668 | 2.797060 | 2.682065 | 0.2874999 | 0.691963 | 1.469406 | 1.374223 | 0.2752639 | 0.8187836 | 0.5639689 | 0.6303045 |
| GluK5 | Control (Vehicle-treated) | 0.7582904 | 1.215308 | 1.085121 | 1.153437 | 1.069317 | 0.8107743 | 0.7947534 | 2.090328 | 0.7175887 | 0.838837 |  |  |
|  | Paclitaxel-treated | 2.561145 | 3.350142 | 2.091332 | 2.472075 | 0.498583 | 1.816052 | 1.809455 | 2.564698 | 2.192048 | 5.506921 | 2.835879 | 4.332588 |

|  |
| --- |
|  |
|  |

**Relative expression of mRNA for kainate glutamate receptors subunits**
