# Supplementary material for: Astrocyte activation in the anterior cingulate cortex and altered glutamatergic gene expression during paclitaxel-induced neuropathic pain in mice
Source: PeerJ. 2015 Oct 22;3:e1350. doi: 10.7717/peerj.1350 (PMC4627912; doi:10.7717/peerj.1350)
Supplement: Supplemental Information 5 [file peerj-03-1350-s005.docx]

| **Receptor** | **Animal number** | **1** | **2** | **3** | **4** | **5** | **6** | **7** | **8** | **9** | **10** | **11** | **12** | **13** | **14** | **15** | **16** |
| --- | --- | --- | --- | --- | --- | --- | --- | --- | --- | --- | --- | --- | --- | --- | --- | --- | --- |
| GluN1 | Control (Vehicle-treated) | 1.319249 | 0.5922179 | 1.009670 | 0.7595412 | 1.028138 | 0.8837335 | 2.005066 | 0.9161358 |  | 0.8917602 | 1.047980 | 1.070037 | 0.7945782 | 0.8261321 | 1.523400 |  |
|  | Paclitaxel-treated | 1.149269 | 1.229141 | 1.272810 | 0.9414676 | 1.086158 | 1.207746 | 0.8522508 | 1.657605 | 1.195000 | 1.764787 | 1.693660 | 1.522168 | 1.530615 | 2.468403 | 3.991215 | 4.078705 |
| GluN2A | Control (Vehicle-treated) | 1.035823 | 1.150062 | 0.839447 | 0.6211011 | 1.140316 | 1.411928 | 1.410885 | 1.162072 | 0.8016253 | 0.7608585 |  |  |  |  |  |  |
|  | Paclitaxel-treated | 1.325813 | 2.129888 | 1.498180 | 1.499375 | 2.388989 | 4.697088 | 3.902414 | 3.032204 | 0.4573699 | 0.8212348 | 0.6534808 | 0.5350825 |  |  |  |  |
| GluN2B | Control (Vehicle-treated) | 0.6876703 | 1.079469 | 1.347130 | 0.5762011 | 1.033982 | 1.678467 | 0.5110415 | 2.197727 | 1.279174 | 0.6960498 |  |  |  |  |  |  |
|  | Paclitaxel-treated | 1.317411 | 2.582149 | 1.615714 | 1.314204 | 1.872689 | 3.254030 | 4.648970 | 5.701248 | 0.3552515 | 0.8572448 | 0.5639852 | 0.5063988 |  |  |  |  |

|  |
| --- |
|  |
|  |

**Relative expression of mRNA for NMDA glutamate receptors subunits**
