# Supplementary material for: Astrocyte activation in the anterior cingulate cortex and altered glutamatergic gene expression during paclitaxel-induced neuropathic pain in mice
Source: PeerJ. 2015 Oct 22;3:e1350. doi: 10.7717/peerj.1350 (PMC4627912; doi:10.7717/peerj.1350)
Supplement: Supplemental Information 6 [file peerj-03-1350-s006.docx]

| **Receptor** | **Animal number** | **1** | **2** | **3** | **4** | **5** | **6** | **7** | **8** | **9** | **10** | **11** | **12** |
| --- | --- | --- | --- | --- | --- | --- | --- | --- | --- | --- | --- | --- | --- |
| mGLuR_1_ | Control (Vehicle-treated) | 2.569997 | 0.3891055 | 1.602627 | 0.795944 | 0.7839438 | 0.6339613 | 1.431175 | 0.9333133 | 1.180911 |  |  |  |
|  | Paclitaxel-treated | 1.763266 | 1.818333 | 2.520162 | 2.613410 | 1.247989 | 1.284766 | 0.8025736 | 0.855045 | 0.4655474 | 1.274767 | 0.8821933 | 0.9989839 |
| mGLuR_2_ | Control (Vehicle-treated) | 1.837019 | 0.5443602 | 1.020525 | 1.069654 | 0.9160793 | 0.8261639 | 1.205933 | 0.9883199 | 1.015577 |  |  |  |
|  | Paclitaxel-treated | 2.931861 | 1.899913 | 2.049398 | 2.902977 | 1.302841 | 0.9641266 | 1.449355 | 0.9363335 | 0.4440877 | 1.087476 | 1.083188 | 0.8721914 |
| mGLuR_3_ | Control (Vehicle-treated) | 2.144810 | 0.4662417 | 0.8603394 | 0.9546504 | 1.217547 | 0.8877959 | 1.036090 | 0.9471074 | 1.147863 |  |  |  |
|  | Paclitaxel-treated | 3.779119 | 3.274444 | 5.547291 | 0.8182675 | 1.688308 | 0.6847952 | 0.5074173 | 0.3851743 | 0.6039495 | 0.5167004 | 0.7202901 |  |
| mGLuR_4_ | Control (Vehicle-treated) | 0.7054477 | 1.050428 | 1.349487 | 0.6964969 | 0.9963599 | 1.441002 | 0.6516978 | 2.488929 | 0.6055319 | 1.018132 |  |  |
|  | Paclitaxel-treated | 0.9070859 | 1.184855 | 0.6416141 | 0.6015866 | 1.465716 | 1.590895 | 0.8891542 | 0.8913345 | 0.4428914 | 1.332521 | 1.008510 | 2.257968 |
| mGLuR_5_ | Control (Vehicle-treated) | 0.5785179 | 1.343441 | 1.286662 | 0.6681194 | 1.021622 | 1.465061 | 0.8229775 | 1.164754 | 0.9585496 | 1.081456 |  |  |
|  | Paclitaxel-treated | 0.9086521 | 1.113036 | 1.029445 | 0.7393349 | 0.9753855 | 2.791669 | 1.866900 | 1.289872 | 0.6217795 | 1.456486 | 0.9544832 |  |
| mGLuR_6_ | Control (Vehicle-treated) | 1.124647 | 2.565261 | 0.3466189 | 0.737393 | 1.160749 | 1.168323 |  |  |  |  |  |  |
|  | Paclitaxel-treated | 0.8005549 | 0.4652535 | 0.7830568 | 1.057389 | 1.816703 | 2.374141 | 0.9178767 | 1.371263 |  |  |  |  |
| mGLuR_7_ | Control (Vehicle-treated) | 0.7509964 | 1.123794 | 1.184883 | 0.909776 | 0.8795678 | 1.249672 | 0.2516626 | 2.759415 | 0.9808895 | 1.468062 |  |  |
|  | Paclitaxel-treated | 1.284385 | 1.841581 | 1.556938 | 1.062192 | 1.246284 | 1.139789 | 0.6308126 | 0.271461 | 1.701113 | 3.385289 | 2.883550 |  |
| mGLuR_8_ | Control (Vehicle-treated) | 0.9161358 | 1.098626 | 0.9935515 | 0.6908154 | 1.002062 | 1.444585 | 0.884637 | 2.006884 | 0.8048308 | 0.6998548 |  |  |
|  | Paclitaxel-treated | 1.298339 | 1.839483 | 1.714772 | 3.193304 | 1.417687 | 2.784701 | 4.335422 | 4.000200 | 0.6762636 | 1.714250 | 1.039787 | 1.311291 |

|  |
| --- |
|  |
|  |

**Relative expression of mRNA for metabotropic glutamate receptors**
