# Supplementary figures and images for: Astrocyte activation in the anterior cingulate cortex and altered glutamatergic gene expression during paclitaxel-induced neuropathic pain in mice
Source: PeerJ. 2015 Oct 22;3:e1350. doi: 10.7717/peerj.1350 (PMC4627912; doi:10.7717/peerj.1350)

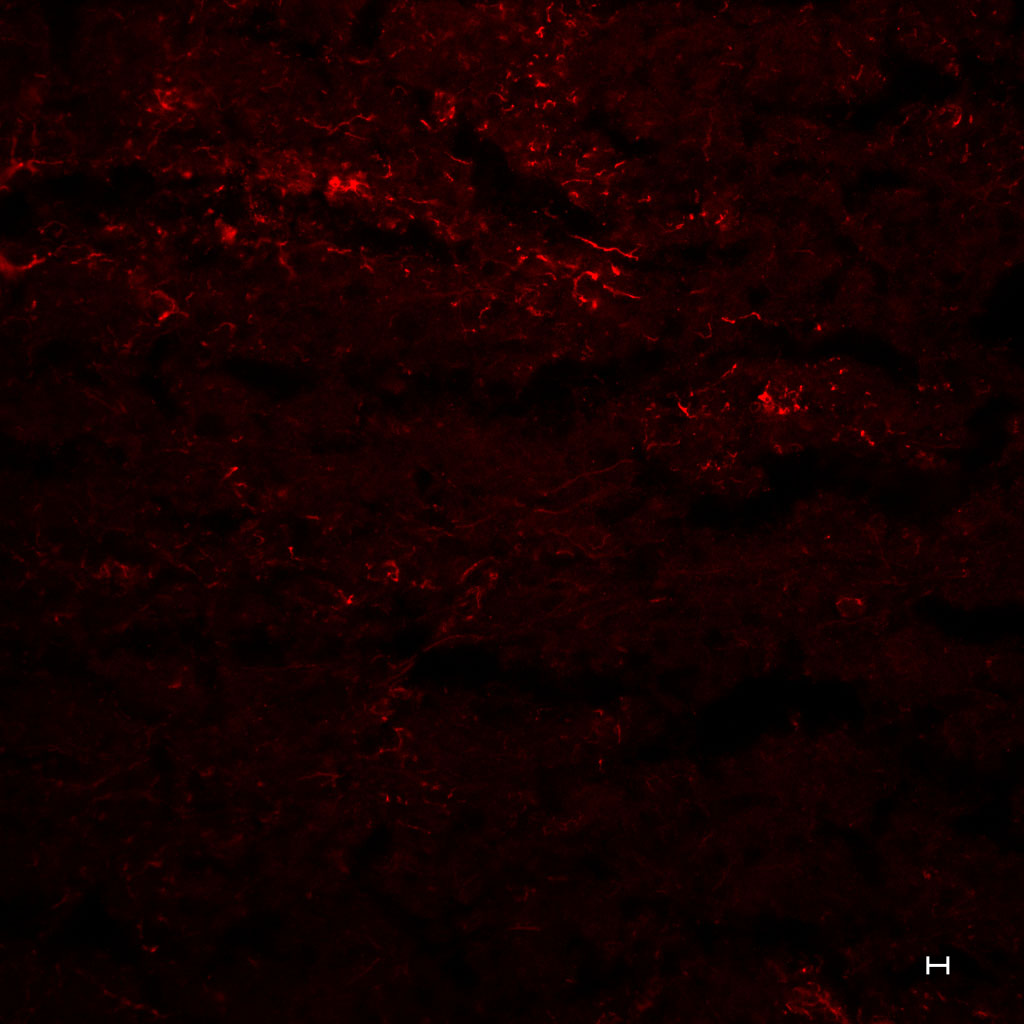

Supplement: Supplemental Information 7 [file peerj-03-1350-s007.jpg]

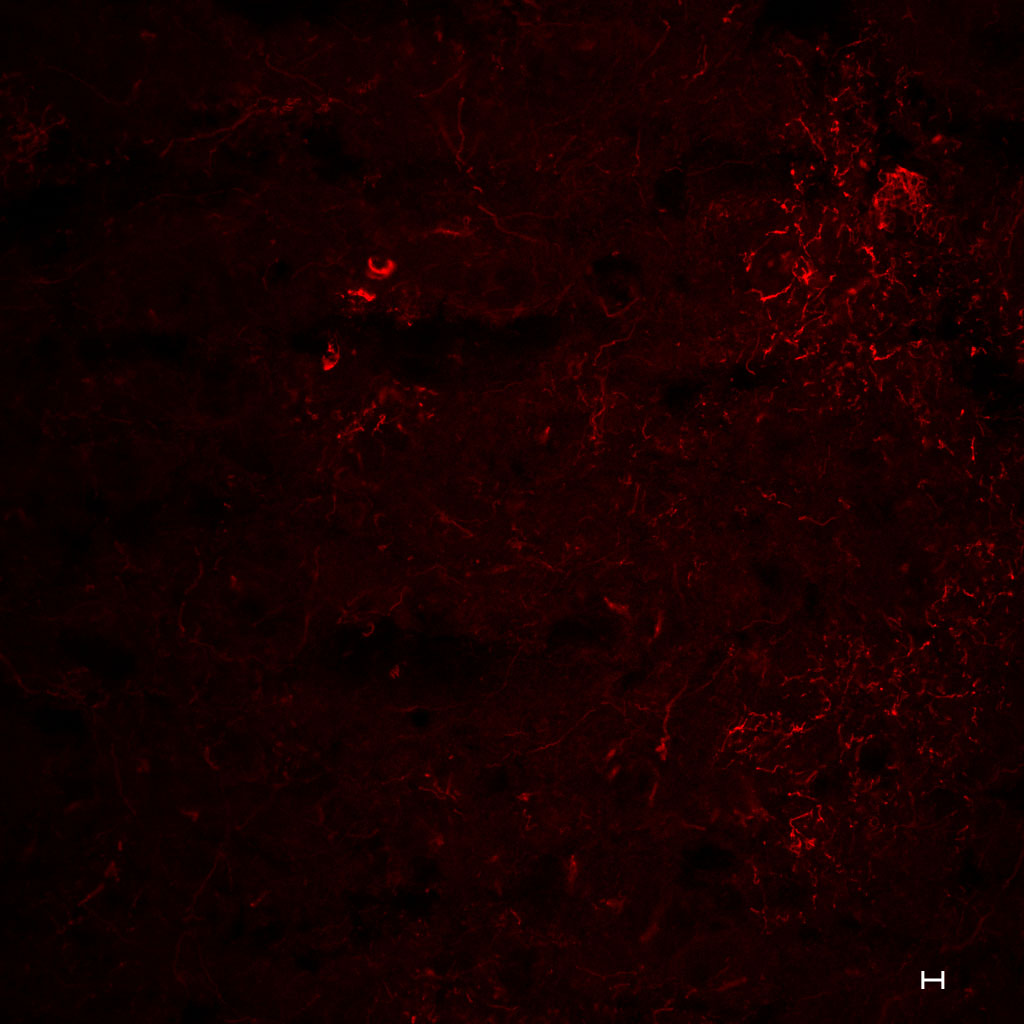

Supplement: Supplemental Information 8 [file peerj-03-1350-s008.jpg]

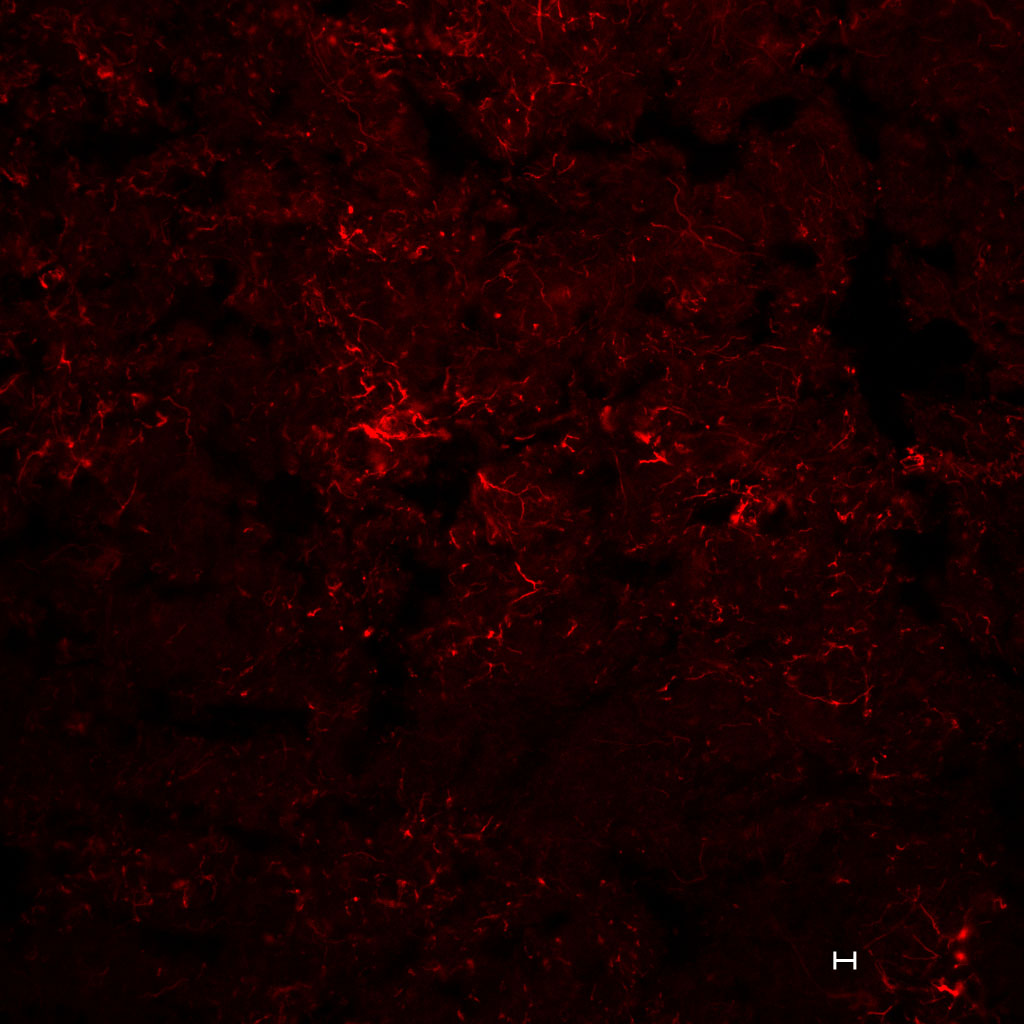

Supplement: Supplemental Information 9 [file peerj-03-1350-s009.jpg]

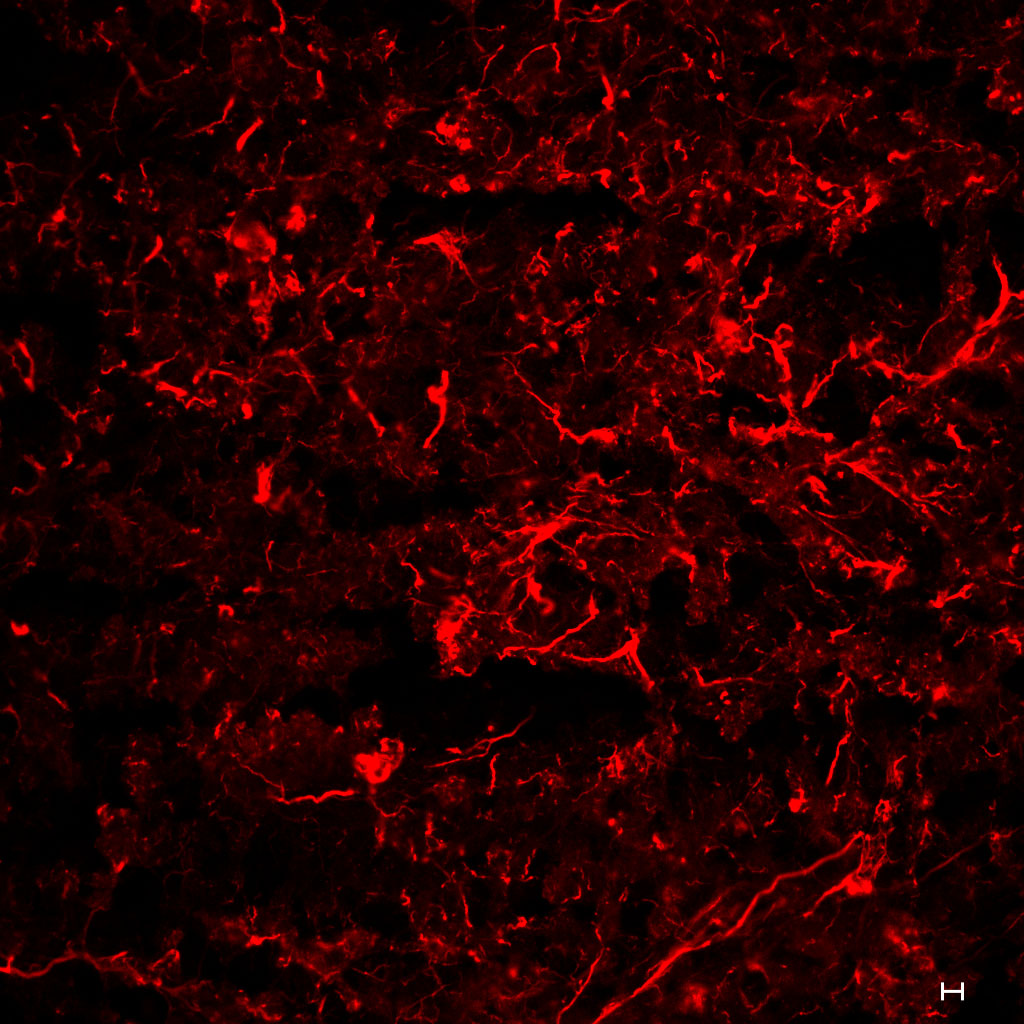

Supplement: Supplemental Information 10 [file peerj-03-1350-s010.jpg]

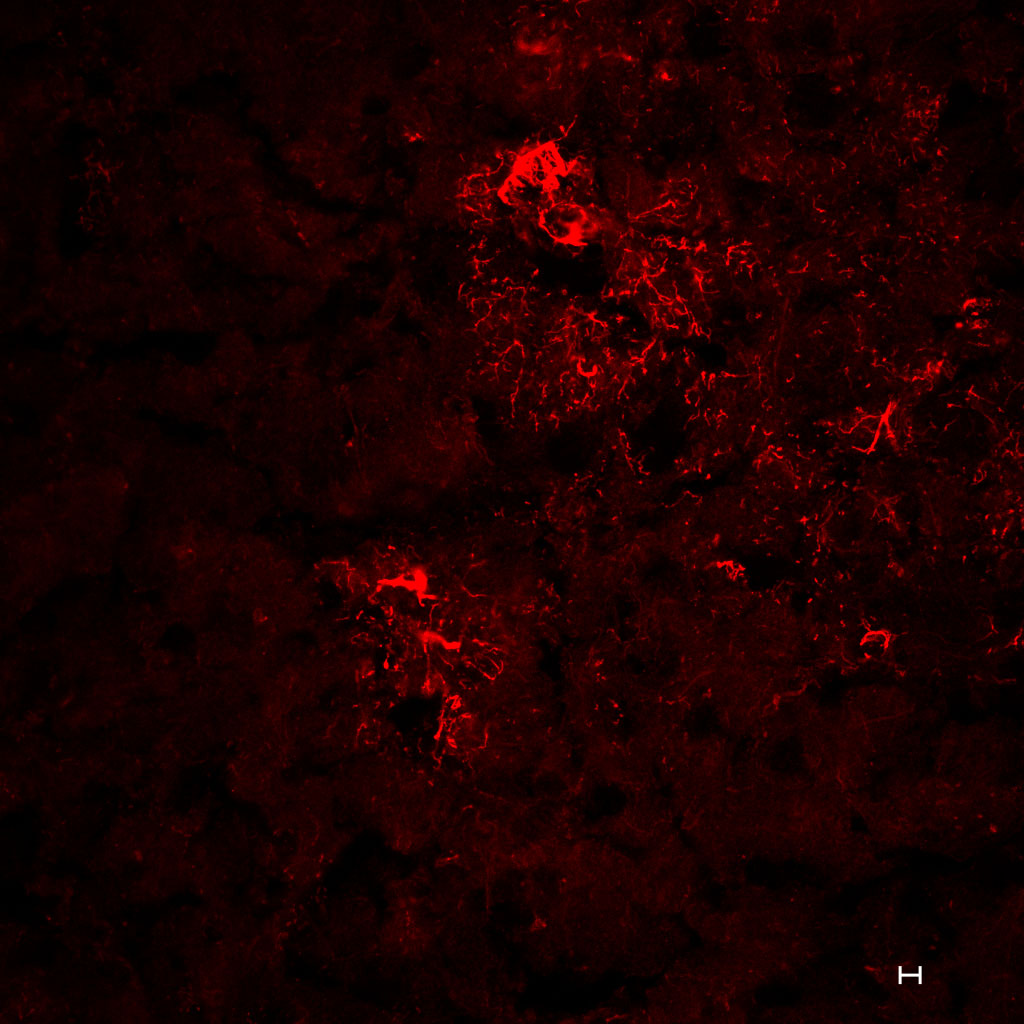

Supplement: Supplemental Information 11 [file peerj-03-1350-s011.jpg]

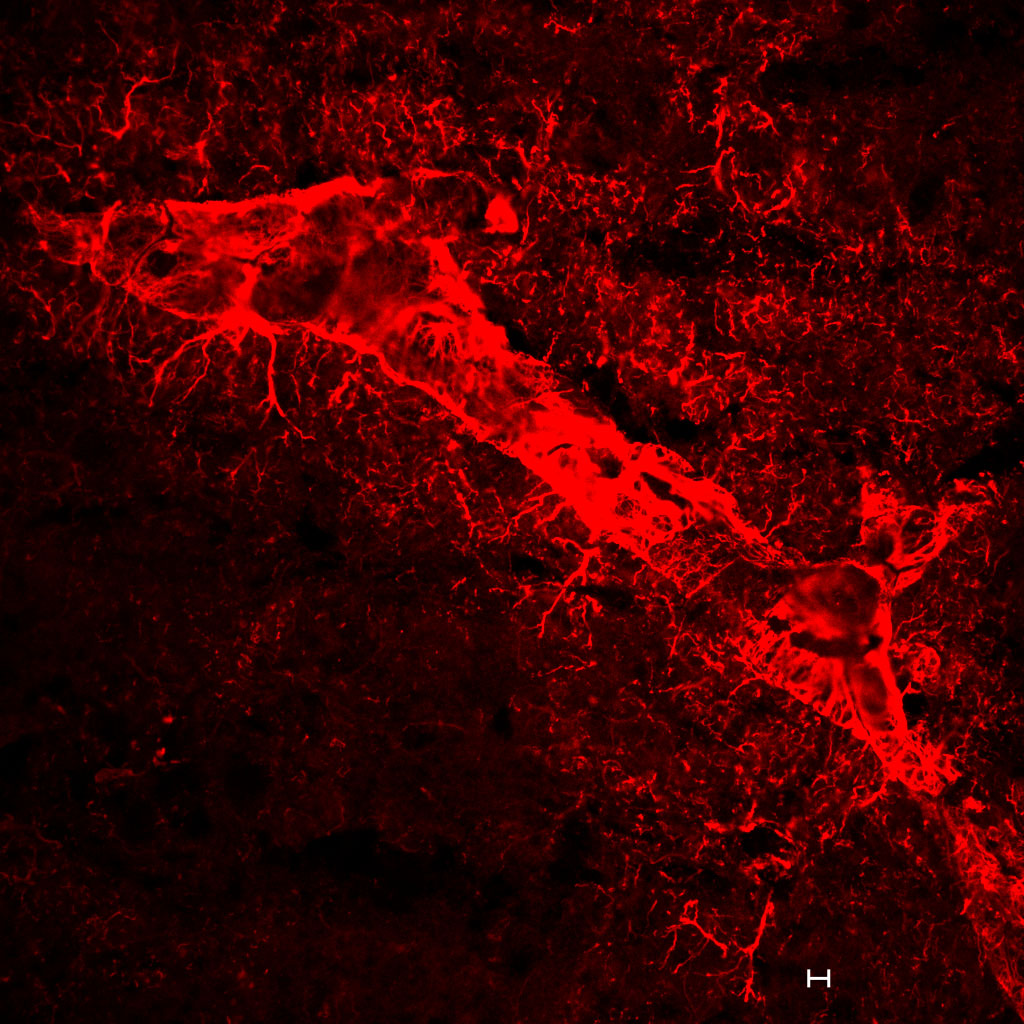

Supplement: Supplemental Information 12 [file peerj-03-1350-s012.jpg]
